# Supplementary material for: HeProMo: A decision support tool to estimate wood harvesting productivities
Source: PLoS One. 2020 Dec 31;15(12):e0244289. doi: 10.1371/journal.pone.0244289 (PMC7774941; doi:10.1371/journal.pone.0244289)
Supplement: Attachment — Submitted filename: PONE-D-20-25452_reviewer_02.pdf [file pone.0244289.s001.pdf]

PLOS ONE

HeProMo: A decision support tool to estimate wood harvesting productivities

--Manuscript Draft--

**Manuscript Number:**	PONE-D-20-25452
**Article Type:**	Research Article
**Full Title:**	HeProMo: A decision support tool to estimate wood harvesting productivities
**Short Title:**	HeProMo: A decision support tool to estimate wood harvesting productivities
**Corresponding Author:**	Stefan Holm<br>Universitat Zurich Institut fur Informatik<br>Zuerich, SWITZERLAND
**Keywords:**	Timber harvesting; productivity model; cost calculation; time calculation; decision support system; open-source software.
**Abstract:**	This work presents a decision support tool that estimates the wood harvesting productivities of 12 different kinds of forest operations under Central European conditions. It includes forest operations using chainsaws, harvesters, skidders, forwarders, chippers, cable and tower yarders, and helicopters. In addition, the tool covers three models for wood volume estimation. The tool is written in Java and available open-source under the Apache License. This work shows how the tool can be used by describing its graphical user interface (GUI) and its application programming interface (API) that facilitates bulk processing of scientific data. Carefully selected default values allow estimations without knowing all input variables in detail. Each model is accompanied by an in-depth documentation where the forest operation, input variables, formulas, and statistical background are given. We conclude that HeProMo is a very useful tool for applications in forest practice, research, and teaching.
**Order of Authors:**	Stefan Holm
	Fritz Frutig
	Renato Lemm
	Oliver Thees
	Janine Schweier
**Additional Information:**	
**Question**	**Response**
**Financial Disclosure**<br><br>Enter a financial disclosure statement that describes the sources of funding for the work included in this submission. Review the submission guidelines for detailed requirements. View published research articles from *PLOS ONE* for specific examples.<br><br>This statement is required for submission and **will appear in the published article** if the submission is accepted. Please make sure it is accurate.	The present work was partially funded by the Swiss Federal Institute for Forest, Snow and Landscape Research (WSL) and the Swiss Federal Office for the Environment (FOEN). The funders had no role in study design, data collection and analysis, decision to publish, or preparation of the manuscript.

Powered by Editorial Manager® and ProduXion Manager® from Aries Systems Corporation

**Unfunded studies**<br>Enter: *The author(s) received no specific funding for this work.*<br><br>**Funded studies**<br>Enter a statement with the following details:<br>• Initials of the authors who received each award<br>• Grant numbers awarded to each author<br>• The full name of each funder<br>• URL of each funder website<br>• Did the sponsors or funders play any role in the study design, data collection and analysis, decision to publish, or preparation of the manuscript?<br>• **NO** - Include this sentence at the end of your statement: *The funders had no role in study design, data collection and analysis, decision to publish, or preparation of the manuscript.*<br>• **YES** - Specify the role(s) played.<br><br>* typeset	
**Competing Interests**<br><br>Use the instructions below to enter a competing interest statement for this submission. On behalf of all authors, disclose any competing interests that could be perceived to bias this work—acknowledging all financial support and any other relevant financial or non-financial competing interests.<br><br>This statement **will appear in the published article** if the submission is accepted. Please make sure it is accurate. View published research articles from *PLOS ONE* for specific examples.	The authors have declared that no competing interests exist.

**NO authors have competing interests**<br><br>Enter: *The authors have declared that no competing interests exist.*<br><br>**Authors with competing interests**<br><br>Enter competing interest details beginning with this statement:<br><br>*I have read the journal's policy and the authors of this manuscript have the following competing interests: [insert competing interests here]*<br><br>* typeset	
**Ethics Statement**<br><br>Enter an ethics statement for this submission. This statement is required if the study involved:<br><br>• Human participants<br>• Human specimens or tissue<br>• Vertebrate animals or cephalopods<br>• Vertebrate embryos or tissues<br>• Field research<br><br>Write "N/A" if the submission does not require an ethics statement.<br><br>General guidance is provided below. Consult the submission guidelines for detailed instructions. **Make sure that all information entered here is included in the Methods section of the manuscript.**	N/A

**Format for specific study types**<br><br>**Human Subject Research (involving human participants and/or tissue)**<br>• Give the name of the institutional review board or ethics committee that approved the study<br>• Include the approval number and/or a statement indicating approval of this research<br>• Indicate the form of consent obtained (written/oral) or the reason that consent was not obtained (e.g. the data were analyzed anonymously)<br><br>**Animal Research (involving vertebrate animals, embryos or tissues)**<br>• Provide the name of the Institutional Animal Care and Use Committee (IACUC) or other relevant ethics board that reviewed the study protocol, and indicate whether they approved this research or granted a formal waiver of ethical approval<br>• Include an approval number if one was obtained<br>• If the study involved *non-human primates*, add *additional details* about animal welfare and steps taken to ameliorate suffering<br>• If anesthesia, euthanasia, or any kind of animal sacrifice is part of the study, include briefly which substances and/or methods were applied<br><br>**Field Research**<br><br>Include the following details if this study involves the collection of plant, animal, or other materials from a natural setting:<br>• Field permit number<br>• Name of the institution or relevant body that granted permission	
**Data Availability**<br><br>Authors are required to make all data underlying the findings described fully available, without restriction, and from the time of publication. PLOS allows rare exceptions to address legal and ethical concerns. See the PLOS Data Policy and FAQ for detailed information.	Yes - all data are fully available without restriction

A Data Availability Statement describing where the data can be found is required at submission. Your answers to this question constitute the Data Availability Statement and **will be published in the article**, if accepted.<br><br>**Important:** Stating 'data available on request from the author' is not sufficient. If your data are only available upon request, select 'No' for the first question and explain your exceptional situation in the text box.<br><br>Do the authors confirm that all data underlying the findings described in their manuscript are fully available without restriction?	
**Describe where the data may be found in full sentences. If you are copying our sample text, replace any instances of XXX with the appropriate details.**<br><br>• If the data are **held or will be held in a public repository**, include URLs, accession numbers or DOIs. If this information will only be available after acceptance, indicate this by ticking the box below. For example: *All XXX files are available from the XXX database (accession number(s) XXX, XXX.*).<br>• If the data are all contained **within the manuscript and/or Supporting Information files**, enter the following: *All relevant data are within the manuscript and its Supporting Information files.*<br>• If neither of these applies but you are able to provide **details of access elsewhere**, with or without limitations, please do so. For example:<br><br>*Data cannot be shared publicly because of [XXX]. Data are available from the XXX Institutional Data Access / Ethics Committee (contact via XXX) for researchers who meet the criteria for access to confidential data.*<br><br>*The data underlying the results presented in the study are available from (include the name of the third party*	The software (executable jar-file) can be downloaded at: https://github.com/holmstefan/hepromo/releases<br>The source code can be accessed at: https://github.com/holmstefan/hepromo

and contact information or URL).<br>• This text is appropriate if the data are owned by a third party and authors do not have permission to share the data.<br><br>* typeset	
Additional data availability information:	

Manuscript

Click here to access/download;Manuscript;PaperHeProMoPlosFormat.docx

HeProMo: A decision support tool to estimate wood harvesting productivities

Stefan Holm [a]*, Fritz Frutig [a], Renato Lemm [a], Oliver Thees [a], Janine Schweier [a]

[a] Swiss Federal Institute for Forest, Snow and Landscape Research WSL, Zürcherstrasse 111, CH-8903 Birmensdorf, Switzerland.

* Corresponding author, Tel.: +41 44 7392 263. Fax: +41 44 7392 215

Email addresses: stefan.holm@wsl.ch, friedrich.frutig@wsl.ch, renato.lemm@bluewin.ch, oliver.thees@wsl.ch, janine.schweier@wsl.ch

Type of Contribution: Research article

Abstract (max 150 words)

This work presents a decision support tool that estimates the wood harvesting productivities of 12 different kinds of forest operations under Central European conditions. It includes forest operations using chainsaws, harvesters, skidders, forwarders, chippers, cable and tower yarders, and helicopters. In addition, the tool covers three models for wood volume estimation. The tool is written in Java and available open-source under the Apache License. This work shows how the tool can be used by describing its graphical user interface (GUI) and its application programming interface (API) that facilitates bulk processing of scientific data. Carefully selected default values allow estimations without knowing all input variables in detail. Each model is accompanied by an in-depth documentation where the forest operation, input variables, formulas, and statistical background are given. We conclude that HeProMo is a very useful tool for applications in forest practice, research, and teaching.

Keywords (maximum of 6 keywords): Timber harvesting, productivity model, cost calculation, time calculation, decision support system, open-source software.

Highlights (3 to 5 bullet points, maximum 85 characters including spaces):

- Tool to estimate harvesting productivities, time, and costs of labor and machinery
- Well-documented models for twelve different kinds of forest operations
- Closes a knowledge-gap for forest operations under Central European conditions
- The models can be accessed via a GUI or API that allows bulk processing
- Written in Java and available open-source under the Apache License

Software Availability:

Name of software: HeProMo

Developer (since version 2014): Stefan Holm (stefan.holm@wsl.ch)

Year First Available: 2000 (COM components) / 2003 (.NET version) / 2014 (Java version)

Programming Language: Java

Requirements: Installed JRE (Java Runtime Environment), minimum version 1.7

Availability: Open source (Apache License 2.0)

The software (executable jar-file) can be downloaded at:

https://github.com/holmstefan/hepromo/releases

The source code can be accessed at:

https://github.com/holmstefan/hepromo

1 Introduction

Sustainable forest management (SFM) has become one of the core concepts in the use and conservation of forest resources, thus in managing forest ecosystems and their services (Schanz, 2004). In general, Ecosystem Services (ES) are the products of functioning ecosystems that benefit people (Deal et al., 2017), and in the field of forestry, one of the most economically important ES is the provision of timber (e.g., round wood, pulp and paper, energy wood). Other ES have regulating (e.g., climate stabilization), cultural (e.g., recreation), and supporting (e.g., nutrient cycling) functions (MEA, 2005). The provision of wood products and other ES depends on various aspects. Because the majority of ES are public goods and they lack of markets, their provision strongly depends on forest ownership structures and political frameworks (Lazdinis et al., 2019; Olschewski et al., 2018).

In this context, the requirements of the society on the forest and the complexity of decision-making in forestry increase. Therefore, the need to calculate the economic effects of forest management in the short, medium, and long term is increasing. Further, forests management strategies significantly influence the amount and quality of provided wood and other ES. When silvicultural prescriptions are implemented, different Forest Operations (FOs) might be more or less favorable for a decision maker. FOs or timber harvesting, which comprises felling, processing, and transport of trees or timber, are responsible for a large part of the costs (approximately 40-60% in Switzerland) and environmental impacts associated to forest management or enterprises. Three common and important harvesting methods in Central Europe include full-tree (FT), tree-length (TL), and cut-to-length (CTL) (Uusitalo, 2010; Labelle and Lemmer, 2019). When applying the FT method, trees are felled and transported to roadsides with branches and top intact. The TL method requires felling, delimbing (removing branches), and topping (cutting the top of the stem at a specified diameter) before skidding to a truck-accessible road. The CTL method involves the same processing as TL, but

with addition of stem bucking (cutting a stem into logs of different specified lengths) before hauling from the stump to a truck-accessible road. All methods can be deployed with different levels of mechanization: motor-manually (MM), semi-mechanized (SM), or fully mechanized (FM).

From a decision maker's perspective, it is essential to estimate working productivity and production costs under given operating conditions before any operation is conducted. This is needed from operating and strategic viewpoints. Therefore, the working time and costs of FOs as well as other aspects such as environmental impacts have to be estimated for entire enterprises or larger regions and an extended period of time.

There are various models to calculate the costs of *individual* harvesting methods and processes (e.g., Ghaffariyan et al., 2013; Triplat & Krajnc, 2020). Some models can also be used to estimate the costs of full supply chains (e.g., Holzernte 8.0 by FVA, 2017). However, these models require productivity to be provided as input data. Further, the models are valid only for a specific region and need to be customized when applied on an enterprise level (e.g., ThüringenForst, 2014). Therefore, this work addresses the lack of a valid collection of models that allows estimating time, productivities, and costs of labor and machinery for the most important FOs in forest stands under Central European conditions. Such a collection could be used as a reference in research, teaching, and practice.

Consequently, our approach was to develop such a tool. In this paper, we aim to

(i) present the open-source software HeProMo[1] and its development from an operational and software engineering perspective;
(ii) demonstrate how it can be used in business and academics for either estimating an individual FO or bulk processing large FO data sets;
(iii) derive future possibilities for the use and development of the software.

The software is a collection of models each representing a certain FO, and it was developed within the last 20 years (Frutig et al., 2009).

2 Methods

HeProMo models represent FOs typically conducted in Central European forests. The forests in these regions are characterized by rather small properties, high share of public owned forests, multifunctional silviculture management as well as mixed and uneven-aged stands. The FOs in these regions are characterized by single tree thinning and comparable small timber volumes per cut, thus resulting in frequent machinery transport and fewer productive machine hours. Further, many restrictions such as soil compaction and nutrient extraction need to be considered. Each HeProMo

[1] "HeProMo" is the abbreviation of "Holzernte-Produktivitäts-Modelle", a German term meaning "wood harvesting productivity models".

model represents a processing or a transport operation – ground-, cable- or air based – and is accompanied by an in-depth pdf-documentation where the FO, input variables, formulas, and statistical background are described. The main output variable calculated by each model is productivity, measured in cubic meters of wood processed per hour. Based on productivity, time and costs of labor and machines are calculated. Costs are displayed as costs per cubic meter and total costs of FO.

The following subsections present an overview of the modeled FOs (section 2.1), data collection process (section 2.2), model building process (section 2.3), and information about the concepts and characteristics of the data units used in HeProMo (section 2.4).

2.1 Modeled forest operations and volume estimations

Table 1 shows an overview of the modeled FOs. In addition to these models, three volume estimators are included in the tool:

- **Biomass estimator**: estimates the volumes of stem wood, compact branch wood, brushwood, and mass of needles and leaves for a set of trees based on tree species and tree location characteristics.
- **Energy wood estimator**: estimates the volumes of energy wood from different parts of a tree, based on the characteristics of a forest area (e.g., proportion of broadleaves, mean diameter at breast height (dbh), and FO (e.g., percentage of logging waste).
- **Stem wood estimator**: estimates the stem volume of a single tree based on tree and location characteristics.

	Model name	Modeled forest operation
Processing Models	Motor-manual felling and processing	Felling, debranching, cutting into logs, and measuring, using a chainsaw.
	Harvester	Felling, processing of trees into logs (debranching, measuring, cutting), and placing branches on the skid trail.
	Chipper	Chipping of crowns and branches, located at a truck-accessible road, into a container.
Mixed Models	Wood chips transport	Transport of wood chips to the customer. Wood chips are either already loaded in a container (lifting system) or chipped into a container mounted on a transport vehicle.
	Felling and pre-skidding	Felling using a chainsaw and pre-skidding of full trees to the skid trail.
Transportation Models	Skidder	Skidding of felled logs from the stand to a storage place at the forest road.
	Forwarder (roundwood only)	Hauling of logs lying at the skid trail (CTL) to a storage place at the forest road.
	Forwarder (roundwood and energy wood)	Hauling of logs and energy wood assortments to a storage place at the forest road. Productivity, time, and costs are differentiated by roundwood and energy wood.
	Long distance cable yarder	Mounting of yarder, yarding of logs from the stand to the forest road using a long distance cable yarder, and dismounting the yarder.
	Tower yarder	Mounting of yarder, yarding of logs from the stand to the forest road using a tower yarder, and dismounting the yarder.
	Tower yarder with mounted processor	Felling by chainsaw, yarding of full trees by tower yarder to the forest road where they are debranched and cut by a mounted processor, and moving logs to a storage place by a separate engine.
	Helicopter	Transportation of logs or full trees from the stand to a truck-accessible road by helicopter.

Table 1: Modeled Forest Operations.

2.2 Data collection

The first digitized productivity models of HeProMo in the early 2000s were derived from existing calculation tools in paper form, such as long distance cable yarder (Abegg et al., 1986). Their data base was founded on manual time studies. Manual time studies can provide detailed information for

an individual case to be recorded. However, they require a corresponding expenditure of time by the personnel who conducted the time studies, in particular where a statistical analysis of data is foreseen. Depending on the demand for representativeness of results, they are associated with high time requirements and costs. In practice, this usually leads to a small amount of data. To overcome the time, quality, and financial constraints, we therefore had to find other methods to obtain data of harvesting processes.

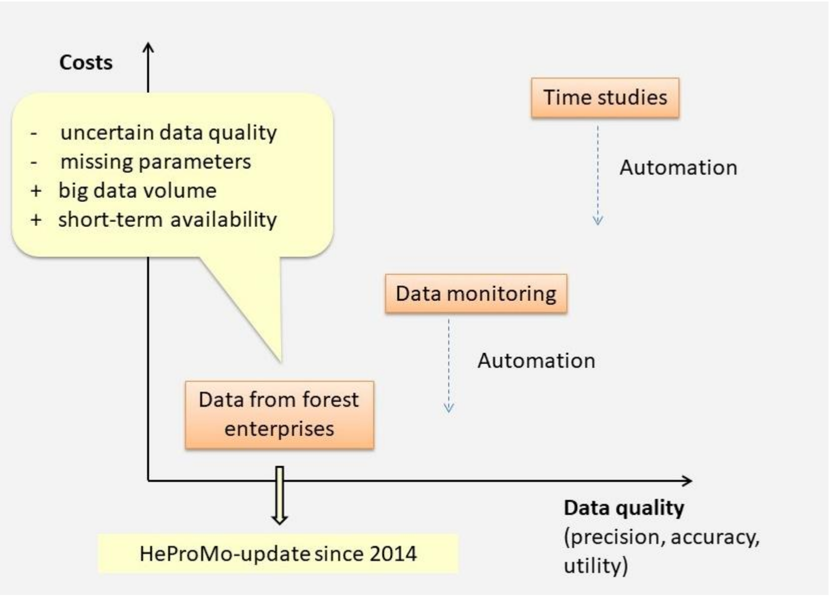

Figure 1: Relation between data quality and costs for different data collection methods (based on Thees & Frutig, 2018).

**Forest operation**	**Data origin**	**Data range**	**Reference**
Motor-manual felling and processing	Forest enterprises	21'879 cuts, 8'000'000 m^3	Bavarian State Forestry Department, Germany
Harvester	Forest enterprises	928 cuts, 9 harvester types, 533'639 m^3	State-owned forestry technical bases (Rhineland-Palatinate and Baden-Wuerttemberg), Germany
Chipper	Publications	5 chipper types, 38 cuts, 4 chipping methods	Kuptz et al. (2015), Germany
Wood chips transport	Publications and small survey	3 transport companies	Hässig (2007), Switzerland
Felling and pre-skidding	Forest enterprises	Tariff d1, combined method with pre-skidding and processing of full trees	State-owned forestry technical base (Baden-Wuerttemberg), Germany
Skidder	Forest enterprises	277 cuts, 5 skidder types, 90'565 m^3	State-owned forestry technical bases (Rhineland-Palatinate and Baden-Wuerttemberg), Germany
Forwarder (roundwood only)	Forest enterprises	336 cuts, 14 machine types, 450'000 m^3	State-owned forestry technical bases (Rhineland-Palatinate and

			Baden-Wuerttemberg), Germany
Forwarder (roundwood and energy wood)	Forest enterprises/publications	Roundwood: 450'000 m^3<br>Energy wood: meta-analysis	Roundwood: State-owned forestry technical bases (Rhineland-Palatinate, Baden-Wuerttemberg, Thuringia), Germany<br><br>Energy wood: Cremer (2008), Germany
Long distance cable yarder	Time studies<br><br>Existing model: Abegg et al. (1986)	44 cuts	44 forest enterprises and private forest operators, Switzerland
Tower yarder	Time studies	22 cable lines, 699 load cycles, ~1800 m^3	22 forest enterprises and private forest operators, Switzerland
Tower yarder with mounted processor	Forest enterprises	104 cuts, 2 machine types, 66'762 m^3	Forest enterprise, Switzerland
Helicopter	Publications	3 helicopter classes	Heinimann (1996, 1998), Switzerland

Table 2: Data sources for different productivity models in HeProMo Version 2.4.

To obtain the data required to update our productivity models in a timely manner with sufficient quality, we used operational data. Hence, most productivity models updated from 2014 are based on data from forest enterprises in Germany and Switzerland (Table 2). Two models were built with data from tests published in forest literature. The productivity models for long distance cable yarder, tower yarder, and helicopter have not yet been updated. Despite being based on rather aged data, there is no urgent need to update these models, as they still fit well.

The procurement of performance data from forest enterprises or service providers is not easy due to competition, obligation of secrecy, and data protection, but also simply due to the lack or restriction of accounting. In general, it is easier to obtain data from public than from private companies. If data are available, they are often large datasets (especially compared to time studies), which allows their statistical analysis. However, the quality of these data cannot be assessed, or only to a limited extent, and performance-determining parameters may be missing from such datasets because the data were collected for other purposes.

As shown in Figure 1, some required parameters may be missing in data sets from forest enterprises because these data have been gathered for uses other than building productivity models. How these cases were handled is described in the next section. Even with some parameters missing, well-fitted productivity models can be produced due to the large data volume (timber volume and time consumption covered by the data).

2.3 Modeling

The development of each HeProMo model follows a uniform construction plan with a three-shell structure (Figure 2). This three-shell structure is important for the flexibility of the use of the models regarding their spatial and temporal context and their maintenance and updating. The core is, in most cases[2], a statistical model, i.e., a regression model of productivity, derived from the available data with the help of the open-source statistical analysis software R[3]. The modeling results are documented and can be tracked by the user. For example, for the skidder model, the influences of average volume, machine type, and logging distance were statistically significant. In the second shell, the selected parameters are known to be relevant for productivity but have not been represented in the given data. Therefore, in this case, the lateral dragging distance, which is another relevant parameter, was implemented as a discount that was estimated by experts from the forest practice. In other cases, we searched for information on influencing factors in the literature or in pre-calculation sheets established by administrations such as German state forest administrations (e.g., influence of skidding or forwarding distances on productivity (ThüringenForst, 2014)). In the third shell, the costs of production factors are introduced into the model.

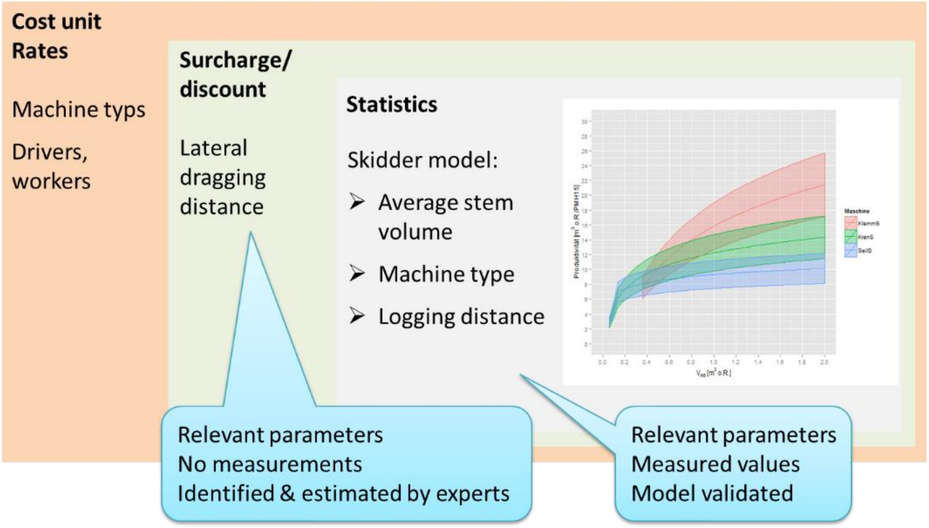

Figure 2: Three-shell structure of the skidder model (based on Thees & Frutig, 2018).

[2] Some models (chipper, woodchips transport, helicopter, volume estimators) are based on information from the literature and expert knowledge, not on an own evaluation of a larger statistical data set.

[3] www.r-project.org

2.4 Data units: concepts and characteristics

2.4.1 Time and productivity

The time system used in HeProMo is based on Björheden & Thompson (1995), modified by Heinimann (1997). Figure 3 presents the used time units and describes their relations among each other.

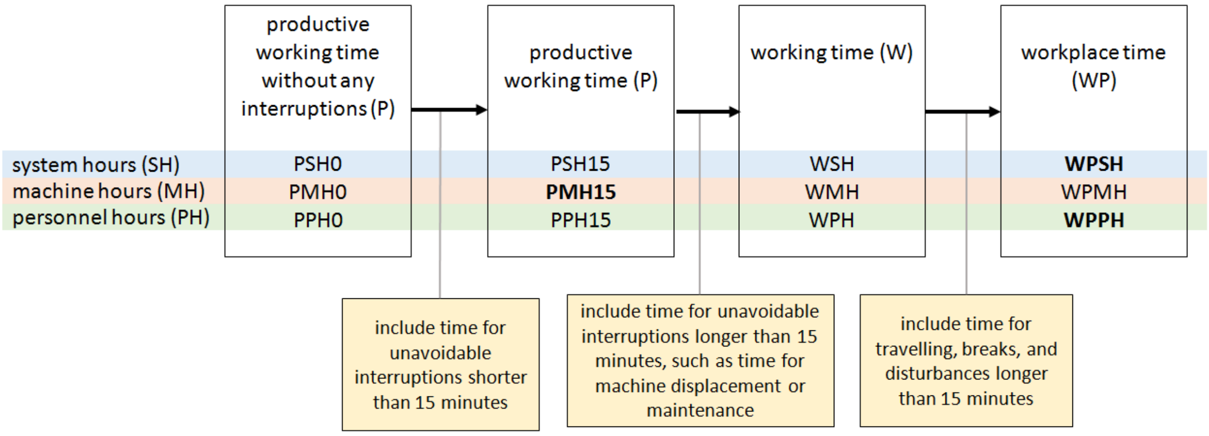

Figure 3: Units used to describe the time of forest operations and their relations among each other. Times can be calculated for the overall system, machinery, or personnel. The most used time units in HeProMo are in bold.

The most relevant time units for HeProMo are the following, which are usually used in the result section of the models:

- *Workplace system hours* (**WPSH**): the work duration, i.e., the time between when the first person starts to work and the last person finishes working.
- *Workplace personnel hours* (**WPPH**): the total time the personnel needs to complete a task ("work time volume"). WPPH may be longer than WPSH if more than one person is working, as WPPH represents the sum of working time of all people working.
- *Productive machine hours* (**PMH15**): the amount of time that a certain machine was running, including interruptions up to 15 minutes (e.g., short maintenance times).

2.4.2 Costs

The cost unit rates for labor and machinery can be manually adapted to local conditions. The default monetary unit for the cost unit rates in HeProMo is Swiss Francs, abbreviated as CHF (1 CHF corresponds to approximately 1 USD as of 2020). Other monetary units such as Euro can also be chosen.

2.4.3 Wood volumes

The most important input variable for all models is the volume of wood to be processed. In most models, this is the merchantable wood volume, reported in cubic meters *over bark*. However, on the

output side, the costs per cubic meter are usually reported *under bark*. This reflects the fact that roundwood is measured over bark but is sold at forest roadside and customers pay the effective under bark volume only. The productivity is reported in both units, under and over bark, with the exception of energy wood related models, which are only reported over bark. The default value for the conversion factor from over to under bark is 0.9 (mean value based on the Swiss National Forest Inventory - NFI) (Brändli, 2010). The conversion factor can be adapted in the user interface.

3 Software

HeProMo is a Java desktop application that can be executed without installation, the only requirement is to have a Java Runtime Environment (JRE) installed on the system. Currently, in 2020, the minimum required JRE version is 1.7. All models can be accessed with a graphical user interface (GUI) and via an application programming interface (API). The source code is available under the Apache License. The models have a varying number of input variables. To facilitate the use of the models, all input variables have reasonable, carefully selected default values. Thus, realistic calculations can be conducted even if some input variables are unknown to the user. Calculations are conducted “on-the-fly”, which means that whenever an input variable is changed, the updated results are immediately shown to the user.

In the following subsections, after a short overview of the history of HeProMo (section 3.1), the access via GUI (section 3.2) and API (section 3.3) are explained, followed by technical information about the software architecture and testing procedure (section 3.4).

3.1 Development history

In 2000, eight productivity models were developed as individual COM[4] software components (Erni et al., 2000a, 2000b), which were incorporated into Excel spreadsheets. In 2003, a .NET application that contained all models within a single piece of software was developed. At the beginning of the 2010s, we decided for an overall update due to the appearance of new harvesting methods and the change in the productivity of FOs since the turn of the millennium. This led to a reimplementation of HeProMo as a Java application, which was made available in 2014. Since then, this version has been continuously maintained and further developed. By 2016, the software was available in English, German, French, and Italian. By 2019, several new models were integrated into the software. These models cover additional FOs or replace already existing models because a newer data base was available. Finally, in 2020, HeProMo was converted into an open-source project, and an API has been

[4] Component Object Model

made available in addition to the GUI. In the following subsections, we present the 2020 version in detail.

3.2 Graphical User interface

3.2.1 Main window

Figure 4 shows the main window of HeProMo. The models are divided into three categories, namely i) "Up-to-date models", ii) "Models with out-dated data base", and iii) "Volume calculation". The "Models with out-dated data base" are available in German only, as they are no longer maintained but still provided to interested users[5]. The other two categories are described in detail in the following sections.

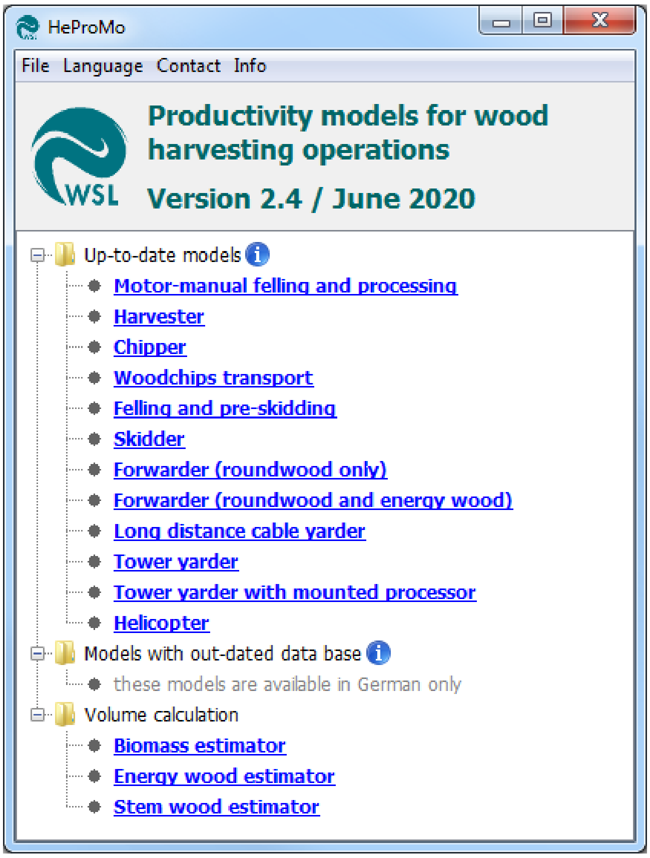

Figure 4: Overview of HeProMo user interface.

[5] These models – legacy versions of the models motor-manual felling and processing, skidder, and forwarder – are only provided in the current model collection of HeProMo because they consist of a higher number of input variables than the new models as from 2014. They may be useful to investigate the influence of certain variables on productivity, even though the level of productivity or cost is outdated. In addition, a number of long-time HeProMo users demand these models and wish them to remain available.

3.2.2 Forest operation models

All models representing FOs have the same structure, which comprises four elements (Figure 5). The first element is a text field in which the current FO can be described. This step is optional but can be useful to identify a specific harvesting site if results are saved or printed out.

The second element is the input area. It consists of the tabs “work item”, “work system”, and “factors”. The “work item” tab contains input parameter fields related to trees, timber, opening-up, and terrain. The most important input field in all models is the volume of wood to be processed. Other input fields are the mean diameter at breast height (dbh), share of conifers, and terrain slope. The second tab, “work system”, consists of input fields for labor and machinery as well as for the related cost unit rates. In addition, input fields for the daily work time can be found there. The “factors” tab allows the change of additional model parameters, such as the conversion factor from over to under bark or a forest-enterprise-specific correction factor to adapt the model to specific conditions. The currency code can be adapted here too.

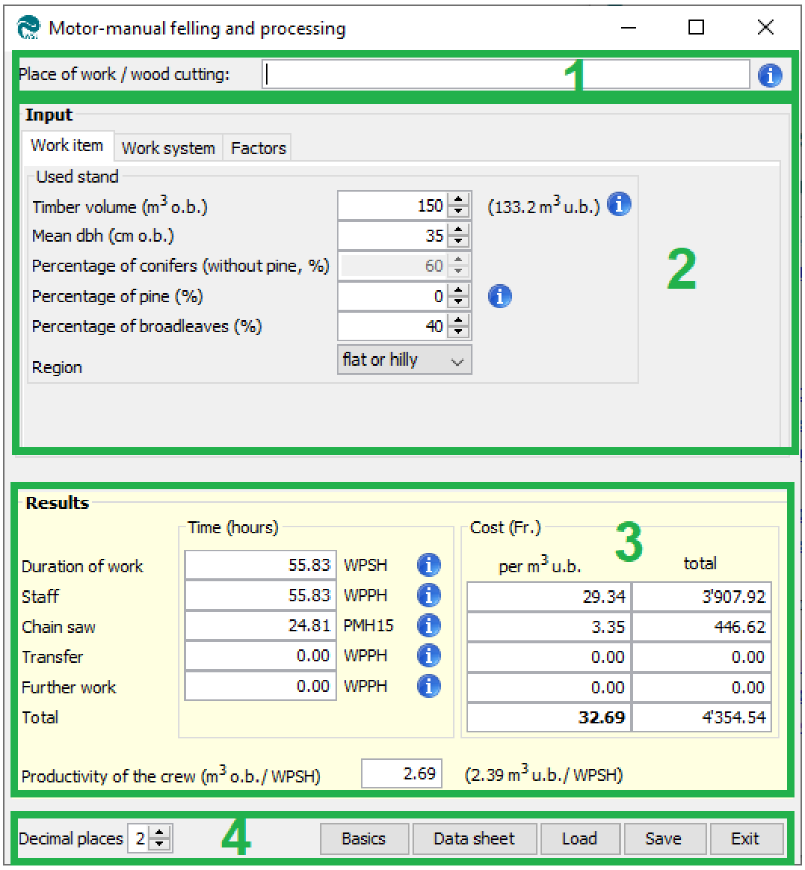

Figure 5: GUI structure of FO models.

The third element is the result section. For all models, it consists of three columns displaying time (c.f. section 2.4.1), costs per cubic meter, and total costs for positions such as labor or machinery.

The forth element consists of the following elements of control:

- Decimal places: allows to adapt the number of decimal places in the results section.
- Basics: opens the documentation of the current model. For most models, the documentation comprises two parts; part A, which describes the model itself (modeled harvesting process, data base, description of input variables, etc.), and part B, which describes and discusses statistics and modeling.
- Data sheet: creates a pdf-file that contains all input variables and the results of the current calculation.
- Save: saves the current input data to a hpm-file.
- Load: reloads input data from a previously saved hpm-file.
- Exit: closes the current model.

Throughout the application, blue info-buttons provide additional information where necessary. Hovering the mouse over such a button displays the additional information in a tooltip.

3.2.3 Volume estimation models

The three models for volume estimations (biomass, energy wood, and stem wood estimator; cf. section 2.1) have a layout similar to that of the FO models. However, they omit separate tabs for the input fields because they have fewer input and output variables (Figure 6).

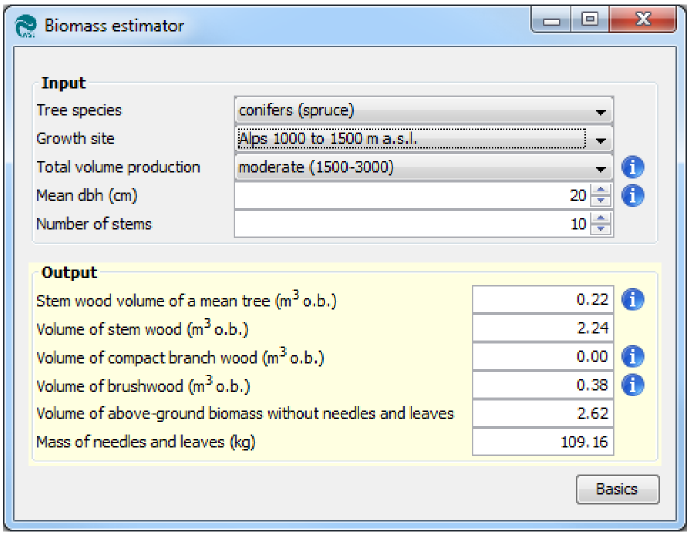

Figure 6: Screenshot of the biomass estimator

3.3 API access

An API allows programmatic access to an application by code of another application. In the last few years, HeProMo was embedded into the code of several other applications, such as applications of the Swiss NFI. Before the API was available, this was done individually for each project with the help

of the HeProMo developer. However, considering the peculiarities of each project, an individual integration is cumbersome to maintain and not sustainable. Therefore, there was a need to create a single well-documented API.

The design of the API focused on five goals: i) clear method names that disclose the precise unit expected or returned, ii) clear documentation of default values, iii) compatibility with programming languages other than Java, iv) performance (high throughput), and v) international usability.

The first goal, clear method names that disclose the precise unit expected or returned, is important because the units of some variables are not self-evident. This includes variables such as volume of wood (over or under bark), transportation distances (meters, kilometers, or miles), cost rates (e.g., of productive worktime or workplace time), and terrain slope (percentage, decimal, or category). This goal was achieved by suffixing each method name with the corresponding unit, e.g., "_ob" for over bark, "_m" for meters, or "_category" for categorical variables.

The second goal, a clear documentation of default values, is important because a default value is assumed for each variable that is not explicitly set. While this behavior is unproblematic in the GUI version because all input variables are directly visible to the user, an API needs a clear documentation of such values to avoid becoming a black-box. This goal was achieved by having a constant in the code defining the default value for each variable. The values of these constants are visible in the Javadoc[6] and are currently the same as in the GUI version.

The third goal, compatibility with programming languages other than Java, is important because the projects that need to access the HeProMo code are written not only in Java but also in languages such as Python or SAS. These languages have a different type system than Java. Therefore, a mechanism to translate types between these languages and Java is needed. In HeProMo, this affects in particular methods with arguments of type *enum*[7], which is used for categorical variables. This goal was achieved by providing for each HeProMo model a dedicated Java class that acts as a wrapper class to the internal code. Such a class has first all the default values defined in constants, followed by all setter- and getter-methods. All methods in these classes use solely the primitive Java type *double* as method argument or return type. This simplifies the use of the class by other programming languages. Therefore, for example, if a method internally needs the terrain slope as an argument of type enum (i.e., the slope is a categorical variable), the API takes a value of type double, rounds it to the closest integer, and translates that integer into the corresponding internal enum value. The

[6] Javadoc is an API documentation in HTML format that can be created directly from the Java source code.
[7] *enum* stands for *enumeration* and is a type of Java class that can be used to define a set of related categorical variables, e.g., "hardwood" and "softwood".

mapping of numbers to categories is described in the Javadoc of the method. Listing 1 shows an example of how the API can be used with Python[8].

The fourth goal, performance, is important because the API is designed to be usable for scientific purposes, where often a large number of calculations must be processed. This goal was achieved by using a lazy-evaluation approach that reduces the number of calculations to a minimum. Setting an input variable does not trigger a calculation, and getting a result variable only triggers a calculation if an input variable has changed since the last calculation. A single calculation provides *all* result variables, as most individual result variables depend on productivity, and calculating the productivity is the computationally most expensive part of the entire calculation process.

The fifth goal, international usability, is important because HeProMo is currently used mainly in German-speaking countries of Central Europe, but the software should also be usable for English-speaking users. This leads to a trade-off regarding the language of the Java class, method, and variable identifiers in the API. All identifiers in the source code were in German so far. Providing only an English API for often highly domain-specific technical terms would be an unnecessary burden for German-speaking API users, as the English translation of well-known technical terms in German is often not straight-forward and can lead to confusion. Therefore, two versions of the API are provided. In addition to an API with German identifiers, we implemented a second version of the API with class, method, and variable identifiers in English for international API users. These classes, which are located in a separate package, were implemented by using the adapter pattern (Gamma et al., 1994) and directly forward calls to the corresponding classes with the German identifiers.

```
# import py4j to connect to the HeProMo-API written in Java
from py4j.java_gateway import JavaGateway

# connect to the JVM
gateway = JavaGateway()

# get a reference to the Java class HeProMoEntryPoint
hepromoEntryPoint = gateway.jvm.ch.wsl.fps.hepromo4python.HeProMoEntryPoint()

# get model for motor-manual felling
model = hepromoEntryPoint.getSimpleMotorManualFellingAndProcessing2014()

# set some input variables
model.setTimberVolume_m3ob(300.0)
model.setMeanDbh_cm(30.0)

# print output
print(model.getProductivity_m3obPerWPSH())
```

Listing 1: Use of the HeProMo-API with Python.

[8] This requires a small helper tool available at https://github.com/holmstefan/hepromo4python/releases/

3.4 Software architecture and testing

The application is completely written in Java and uses Swing as the GUI toolkit. The source code is divided into four packages, namely hepromo.api, hepromo.gui, hepromo.model, and hepromo.util. The model package contains the core functionality and should not be directly accessed by external code as its interface may change - in contrary to the api package, which is designed to remain stable. The model package is completely independent of the gui and api packages. Therefore, the software could be distributed without the gui or without the api package if necessary. The classes in the model package follow closely the presentation of the models in the GUI, i.e., each model has a class for work item, work system, and factors. Further, each model has a calculator-class that transforms the input variables into a result-object containing all output variables. To create pdf files, the Apache FOP library[9] is used.

All models have been tested using TestNG[10] by applying a data-driven testing (DDT) approach. There is a csv-file for each HeProMo model containing the test cases for that model. This csv-file contains a test case on each row: the first columns contain input variables, and the later columns the expected output variables. With this approach, approximately 25’000 output values are verified whenever the test suite is run.

In addition, AssertJ Swing[11] is used to perform the GUI tests. The goal of these tests is to assure that the unit of an output value displayed in the GUI corresponds to the unit that was actually calculated for that value. This was introduced because the units (e.g., for the harvesting productivity) sometimes slightly differ across different models.

4 Discussion

4.1 Business use

For business use, such as for a one-time estimation of time and costs for a single stand before realizing the FO, the use of the GUI version of HeProMo is recommended.

As all input fields have a carefully selected default value, a preliminary calculation result is already shown when the FO model is opened in the application. However, two kinds of input variables need to be adapted in most cases: those specific to the FO site and those specific to the company.

[9] https://xmlgraphics.apache.org/fop/
[10] https://testng.org
[11] https://joel-costigliola.github.io/assertj/assertj-swing.html

The most important input variable related to the FO site is the volume of wood to be harvested or processed. Additional input values such as mean dbh, share of conifers, and terrain slope increase the accuracy of the productivity estimation.

Values such as cost unit rates of labor and machinery are company-specific. Therefore, these values should be adapted and saved, so they can be loaded again the next time HeProMo is used. Another company-specific value is the *specific correction factor*, which can be set in each model. This factor allows the model to be adjusted for company-specific conditions, for example, if a company has a systematically lower or higher productivity for a certain FO due to slightly deviating harvesting methods or the use of different machinery.

Input fields should be self-explanatory. In case of possible ambiguities of input variables, there is an info button next to the input field. The equal input and output field structure in all models simplifies the use of the software. Finally, all models are accompanied by a manual, where all the basics and mathematics behind the model are documented in detail.

4.2 Scientific use

Increasing scientific applications of HeProMo are a result of the growing need to quantify the sustainability impacts of wood use. For this purpose, HeProMo is a suitable tool to estimate both monetary and environmental impacts of timber harvesting. For the latter, it can provide the basis to relate emissions and resource consumption of FOs to their performance. Therefore, HeProMo can be applied in life cycle assessments (e.g., Berendt et al., 2018) and has been used to develop forestry standards that are used in the life cycle inventory database ECOINVENT (Werner, 2017). The applications for scientific studies and in the context of demanding practical applications are mostly of a more complex nature and require the processing of bulk data.

4.2.1 Example of bulk processing

Contrary to business use, scientific use of such models often require the estimation of not only a single FO, but a large set of different FOs with varying input parameters. This is done, for example, in the context of the Swiss NFI (Brändli, 2010; Fischer & Traub, 2019). Within NFI, HeProMo is used to estimate timber harvesting costs for NFI sample plots (Fischer & Stadelmann, 2019), for the simulation of forest management scenarios (Stadelmann et al., 2019a, 2019b), and in further ongoing projects. For this kind of applications, a possibility to access the HeProMo models programmatically is essential.

However, it was difficult to integrate the earlier version of HeProMo programmed in .NET into the NFI applications written in SAS. Therefore, the core algorithms of HeProMo needed to be translated into SAS code (Fischer & Stadelmann, 2019). With the new Java-HeProMo and its API, both projects

written in Java and in other languages, such as SAS or Python, can now directly access the HeProMo code through its API. This emphasizes the value of the new HeProMo implementation, as the clearly documented API facilitates the programmatic use of HeProMo.

4.2.2 Examples of international use

A literature review revealed that published studies referring to HeProMo currently (2020) mainly cover study regions in Switzerland. However, a few studies from other countries and research institutes have been identified (Table 3). Five out of seven studies were conducted in German-speaking countries. Since 2016, HeProMo is available not only in German but also in English, French, and Italian, which is expected to facilitate its use in other countries.

Reference	Study region	HeProMo version used	HeProMo models used
Schweier & Ludowicy (2020)	German	Java version	Motor-manual, forwarder, skidder
Berendt et al. (2018)	Germany	Java version	Harvester, forwarder
Englisch et al. (2016)	Austria	.NET version	(Harvester, skidder, chipper, cable cranes)
Di Fulvio et al. (2016)	European Union (EU28)	Directly used formulas from documentation	Motor-manual felling with chainsaw
Nemestothy et al. (2014)	France, Italy, Slovenia	.NET version and Excel version	Cable cranes (harvester, forwarder)
Cremer (2008)	Germany	.NET version	Forwarder, skidder, chipper. (motor-manual, harvester, cable cranes, helicopter)
Neumann & Schadauer (2007)	Austria	.NET version	Motor-manual, cable cranes

Table 3: Studies using HeProMo for study regions outside Switzerland. A model name in brackets indicates that the model is either not explicitly mentioned but assumedly used or that the model is mentioned but it is uncertain if it was actually used.

4.3 Other software for estimating productivities and costs

Few software tools that allow for a direct estimate of wood harvesting productivities are described in the literature. Examples of such tools are the STHARVEST (Fight et al., 2003), IRLPACA (Murphy et al., 2010), and HCR Estimator (Becker et al., 2008, 2009). However, none of them were developed for Central European conditions. Another category of tools (e.g., Kostadinov et al., 2012; Holm, 2019) use the k-nearest-neighbors-algorithm to estimate FO productivities (Lemm et al., 2005). This

algorithm is especially suitable for forest enterprises and private forest owners to estimate productivities based on their own data records of former FOs.

In addition to such software tools, numerous scientific articles present formulas to calculate productivities and in most cases also costs for all kinds of FOs and under various conditions (e.g., Sirén & Aaltio, 2003; Kärhä et al., 2004; Jiroušek et al., 2007; Eriksson & Lindroos, 2014). A cross-section and comparison of 27 studies of this kind can be found in Hiesl and Benjamin (2013). However, these software tools and formulas are always specific to particular FOs and conditions. Mostly, they refer to FOs using CTL systems with harvesters and forwarders. HeProMo complements the existing tools by allowing productivity estimation of 12 kinds of FOs under Central European conditions. In particular, there are motor-manual processes included, which are still often used in the Central European Alps due to the terrain conditions.

4.4 Environmental relevance

In addition to economic aspects, performance indicators have been increasingly focusing on energetic inputs and greenhouse gas (GHG) emissions of FOs to enhance their environmental performance (e.g., Athanassiadis, 2000; Klvac & Skoupy, 2009; Lijewski et al., 2017). The most recent and comprehensive reviews were provided by Klein et al. (2015), Cosola et al. (2016), and Đuka et al. (2017). Most emissions caused by FOs depend on the fuel consumption of machines (e.g., Pandur et al., 2015), and fuel consumption strongly correlates with machine productivity (e.g., Nordfjell et al., 2003; Freitas, 2004; Klvac & Skoupy, 2009). Therefore, HeProMo can be used to optimize processes from an environmental viewpoint once the FO productivity is known.

4.5 Limitations

There are some constraints in using the HeProMo productivity models. Due to the data base, each model is built for a specific harvesting process. In practice, harvesting processes can occur under conditions (terrain, machinery, operational procedure) that do not correspond exactly to the conditions of the FOs used for modeling.

Often, forest enterprises and private forest operators try to perfect the harvesting processes, e.g., by using additional machinery. If so, the productivity model can be adapted to their needs by using a specific correction factor (see 4.1).

The HeProMo productivity models are based on data of FOs in Central European forests, mainly in Switzerland and Germany. There, FOs occur in forests treated by specific sylvicultural methods. The main characteristics are single tree harvesting, mixed and uneven-aged stands (conifer and broadleaves), and many environmental restrictions, e.g., soil protection on skid trails. Therefore, for

other conditions such as clear-cuts and few environmental restrictions, the productivity model would not fit best.

4.6 Outlook

The current HeProMo is the result of significant developing efforts over the past two decades. To prevent the software from becoming outdated and the loss of invested work, continuous maintenance and improvement of the software are essential. Possible directions for the future of HeProMo are outlined in the following paragraphs.

In addition to the continuous general maintenance of the software, it is planned to continuously further develop HeProMo by adding additional models or adapting existing ones whenever necessary. The goal of going open-source is to support such enhancements and make the software even more transparent. It offers the opportunity for everyone to extend HeProMo, for example, by adding additional models, fine-tuning existing ones according to the user's needs, or translating the application into other languages. Finally, it mitigates the risk of the source code of this long-standing project getting lost over the years.

As the basis of an empirical model is statistical data, collecting up-to-date data is an important prerequisite for the future development of HeProMo. Updating the models is often difficult because there are no data available from forest enterprises or forest operators. In the future, data collection processes could be improved by establishing a permanent monitoring system not yet installed and automated data-collecting methods.

Finally, in the future, HeProMo could be extended vertically and horizontally. Vertically, by integrating relevant ecological effects of FOs into the models (in addition to productivity, time, and costs). Horizontally, by integrating HeProMo into a library of models covering not only harvesting processes, but also processes related to planting, tending of young forest and forest stands, and forest protection.

5 Conclusions

This article introduced HeProMo, an open-source software to estimate the wood harvesting productivities of 12 different kinds of forest operations under Central European conditions. Having a collection of models with a common structure and the possibility of a programmatic access via an API is an important prerequisite for the calculation of productivities and costs of forest operations in scientific applications such as in a national forest inventory. While forest enterprises could rely on their experience to estimate their individual productivities, this is not an option for scientific applications, which need to calculate according to a common standard. Nevertheless, even for forest enterprises and practitioners, HeProMo offers new possibilities to enhance their processes by

allowing estimations of forest operations before they are realized, with a tool that has a solid data base and is easy to use.

After HeProMo evolved and was proven useful in science and industry for more than 20 years, it is now being made open-source. By making the code available to the scientific community and interested practitioners, we lay the foundation for further continuous improvement of the software and its use in a broad range of applications in science and practice.

6 Acknowledgements

As the development of HeProMo is an ongoing project that started two decades ago, there have been multiple sources of funding over the years. The present work was partially funded by the Swiss Federal Institute for Forest, Snow and Landscape Research (WSL) and the Swiss Federal Office for the Environment (FOEN).

In addition, the authors would like to thank all data suppliers (cf. Table 2), without whom it would not have been possible to create such a collection of forest operation models.

7 References

Abegg, B., Frutig, F., Wüthrich, W., 1986. Kalkulationsunterlagen für den Seilkraneinsatz. Merkblatt für den Forstpraktiker Nr. 11 [Calculation documents for the use of a cable crane. Instruction sheet for the forestry practitioner No. 11]. Eidg. Anstalt für das forstliche Versuchswesen, Birmensdorf. 8 S.

Ackerman, P., Belbo, H., Eliasson, L., de Jong, A., Lazdins, A., Lyons, J., 2014. The COST model for calculation of forest operations costs. IJFE, 25(1), 75-81. https://doi.org/10.1080/14942119.2014.903711.

Athanassiadis, D., 2000. Energy consumption and exhaust emissions in mechanized timber harvesting operations in Sweden. Sci Total Environ 255 (1-3), 135–143. DOI: 10.1016/S0048-9697(00)00463-0.

Becker, D. R., Larson, D., Lowell, E. C., & Rummer, R. B., 2008. User guide for HCR Estimator 2.0: software to calculate cost and revenue thresholds for harvesting small-diameter ponderosa pine. Gen. Tech. Rep. PNW-GTR-748. Portland, OR: US Department of Agriculture, Forest Service, Pacific Northwest Research Station. 51 p., 748.

Becker, D. R., Larson, D., & Lowell, E. C., 2009. Financial considerations of policy options to enhance biomass utilization for reducing wildfire hazards. Forest Policy and Economics, 11(8), 628-635.

Berendt, F., Fortin, M., Suchomel, C., & Schweier, J., 2018. Productivity, costs, and selected environmental impacts of remote-controlled mini forestry crawlers. Forests, 9(10), 591.

Bjorheden, R., & Thompson, M. A., 1995. An international nomenclature for forest work study. In: Proceedings of IUFRO 1995 S3:04 Subject Area: 20th World Congress; Tampere, Finland. Orono, Maine: University of Maine: 190-215.

Brändli, U. B. (Ed.)., 2010. Schweizerisches Landesforstinventar: Ergebnisse der dritten Erhebung: 2004-2006 [Swiss National Forest Inventory: Results of the third survey: 2004-2006]. Swiss Federal Research Institute (WSL) and Federal Office for the Environment (FOEN). 312 p. (in German).

Cosola, G., Grigolato, S., Ackerman, P., Monterotti, S., Cavalli, R., 2016. Carbon Footprint of Forest Operations under Different Management Regimes. Croat. J. For. Eng. 37 (1), 201–217.

Cremer, T., 2008. Bereitstellung von Holzhackschnitzeln durch die Forstwirtschaft. Produktivitätsmodelle als Entscheidungsgrundlage über Verfahren und Aushaltungsvarianten, entwickelt auf der Basis einer Metaanalyse [Provisioning of wood chips by the forestry. Productivity models as a decision basis for processes and bucking variants, developed on the basis of a meta analysis]. Dissertation. Albert-Ludwigs-Universität, Freiburg i. Brsg., 199 S.

Deal, R., L., Smith, N., Gates, J., 2017. Ecosystem services to enhance sustainable forest management in the US: moving from forest service national programmes to local projects in the Pacific Northwest Forestry, 90 (5), 632-639. https://doi.org/10.1093/forestry/cpx025

Di Fulvio, F., Forsell, N., Lindroos, O., Korosuo, A., & Gusti, M., 2016. Spatially explicit assessment of roundwood and logging residues availability and costs for the EU28. Scandinavian Journal of Forest Research, 31(7), 691-707.

Đuka, A., Vusić, D., Horvat, D., Šušnjar, M., Pandur, Z., Papa, I., 2017. LCA Studies in Forestry – Stagnation or Progress? Croat. J. For. Eng. 38 (2), 311–326.

Englisch, M., Gschwantner, T., Ledermann, T., & Katzensteiner, K., 2016. An Integrated Approach to Assess Sustainable Forest Biomass Potentials at Country Level. Biochar, 123.

Eriksson, M., Lindroos, O., 2014. Productivity of harvesters and forwarders in CTL operations in northern Sweden based on large follow-up datasets. IJFE 25 (3), 179–200. DOI: 10.1080/14942119.2014.974309.

Erni, V., Frutig, F., Lemm, R., Oswald, K., Thees, O., 2000a. Produktivitätsmodelle für Verjüngung, Jungwaldpflege und Holzernte mit Hilfe komponentenbasierter Softwaretechnologie [Productivity models for regeneration, young forest maintenance, and harvesting with the help of component-based software technology]. Schlussbericht zum Projekt Nr. 98.03 des Wald- und Holzforschungsförderungsfonds [Final report of project nr. 98.03 of the forest and wood research promotion fund]. Swiss Federal Institute for Forest, Snow and Landscape Research WSL. Unpublished. 57 p.

Erni, V., Frutig, F., Lemm, R., Oswald, K., Thees, O., 2000b. Produktivitätsmodelle für Verjüngung, Jungwaldpflege und Holzernte mit Hilfe komponentenbasierter Softwaretechnologie [Productivity models for regeneration, young forest maintenance, and harvesting with the help of component-based software technology]. Schlussbericht zum Projekt Nr. 98.03 des Wald- und Holzforschungsförderungsfonds [Final report of project nr. 98.03 of the forest and wood research promotion fund]. Zusammenstellung der Grundlagen für die Programmierung [Report of the basics for the software development]. Swiss Federal Institute for Forest, Snow and Landscape Research WSL. Unpublished. 190 p.

Fight, R. D., Zhang, X., & Hartsough, B. R., 2003. Users guide for STHARVEST: software to estimate the cost of harvesting small timber. Gen. Tech. Rep. PNW-GTR-582. Portland, OR: US Department of Agriculture, Forest Service, Pacific Northwest Research Station. 12 p, 582.

Fischer, C., & Stadelmann, G., 2019. Calculation of Potential Timber Harvesting Costs (HeProMo). In Swiss National Forest Inventory–Methods and Models of the Fourth Assessment (pp. 257-263). Springer, Cham.

Fischer, C., & Traub, B. (Eds.), 2019. Swiss National Forest Inventory - Methods and Models of the Fourth Assessment. Managing forest ecosystems, 35. 431 p. Springer.

Freitas, C., 2004. Análise dos encargos horários com a utilizacao das máquinas pesadas na agricultura e floresta [Analysis of the working times associated with the use of heavy machinery in agriculture and forestry]. Lisboa, Protugal: IHERA Gabinete de Gestao do Parque de Maquinas, Direccao de Servicos de Projectos e Obras, Instituto de Desenvolvimento Rural e Hydraulica.

Frutig, F., Thees, O., Lemm, R., Kostadinov, F., 2009. Holzernteproduktivitätsmodelle HeProMo – Konzeption, Realisierung, Nutzung und Weiterentwicklung [Timber harvesting productivity models HeProMo - conception, realization, utilization and further development]. In: Management zukunftsfähige Waldnutzung. Grundlagen, Methoden und Instrumente. Zürich: VDF, 441-466.

FVA, 2017. Holzernte 8.0 [timber harvesting 8.0]. https://www.fva-bw.de/daten-und-tools/tools/holzernte-80.

Gamma, E., Helm, R., Johnson, R., Vlissides, J., 1994. Design Patterns: Elements of Reusable Object-Oriented Software. Addison Wesley. pp. 139ff. ISBN 0-201-63361-2.

Ghaffariyan, M. R., Spinelli, R., Brown, M., Mirowski, L., 2013. A model to predict productivity of different chipping operations. South Forests 75 (3), 129–136. http://dx.doi.org/10.2989/20702620.2013.816233.

Hässig, J., 2007. Produktivität und Kosten beim Transport von Rundholz, Industrieholz, Energieholz und Hackschnitzeln [Productivity and costs in the transport of round timber, industrial wood, energy wood and wood chips]. Internal Excel calculation sheet, unpublished. Eidg. Forschungsanstalt WSL..

Heinimann, H.R., Caminada, L., 1996. Helicopter Logging in Switzerland. Analysis of Selective Logging Operations. Proceedings 9th Pacific Northwest Skyline Symposium: 40–45. FERIC Special Report SR-114, Vancouver, 167 S.

Heinimann, H.R., 1997. An International Nomenclature For Forest Work Study, Swedish University of Agricultural Sciences, Department of Operational Efficiency, Sweden: Skript Forstl. Verfahrenstechnik, ETH Zürich.

Heinimann, H.R., 1998. Holzrücken mit Helikoptern [timber extraction with helicopters]. Wald & Holz, 79(3):7-10.

Hiesl, P., & Benjamin, J. G., 2013. Applicability of international harvesting equipment productivity studies in Maine, USA: A literature review. Forests, 4(4), 898-921.

Holm, S., 2019. WSL-kNN: Neue Software zur Schätzung des Holzernteaufwandes [new software to estimate wood harvesting costs]. https://www.waldwissen.net/technik/holzernte/kalkulation/wsl_knn_schaetzung_holzernteaufwand/index_DE.

Jiroušek, R., Klvač, R., Skoupý, A., 2007. Productivity and costs of the mechanised cut-to-length wood harvesting system in clear-felling operations. J For Sci 53 (10), 476–482.

Kärhä, K., Rönkkö, E., Gumse, S.-I., 2004. Productivity and Cutting Costs of Thinning Harvesters. IJFE 15 (2), 43–56. DOI:10.1080/14942119.2004.10702496.

Klein, D., Wolf, C., Schulz, C., Weber-Blaschke, G., 2015. 20 years of life cycle assessment (LCA) in the forestry sector. State of the art and a methodical proposal for the LCA of forest production. Int J Life Cycle Assess 20 (4), 556–575. DOI: 10.1007/s11367-015-0847-1.

Klvac, R., Skoupy, A., 2009. Characteristic fuel consumption and exhaust emissions in fully mechanized logging operations. J For Res 14 (6), 328–334. DOI: 10.1007/s10310-009-0143-7.

Kostadinov, F., Lemm, R., Thees, O., 2012. A software tool for the estimation of wood harvesting productivity using the kNN method. Schweizerische Zeitschrift für Forstwesen, 163(4), 119-129.

Kuptz, D., Schulmeyer, F., Hüttl, K., Dietz, E., Turowski, P., Zormaier, F., Borchert, H. und Hartmann, H., 2015. Optimale Bereitstellungsverfahren für Holzhackschnitzel [optimal supplying of wood chips]. LWF Bayerische Landesanstalt für Wald und Forstwirtschaft. 316 S.

Labelle, E., Lemmer, K.J., 2019. Selected Environmental Impacts of Forest Harvesting Operations with Varying Degree of Mechanization. CROJFE 40 (2). doi: https://doi.org/10.5552/crojfe.2019.537.

Lazdinis, M., Angelstam, P., Pülzl, H., 2019. Towards sustainable forest management in the European Union through polycentric forest governance and an integrated landscape approach. Landscape Ecol, 2–13. https://doi.org/10.1007/s10980-019-00864-1.

Lemm, R., Vogel, M., Felber, A., & Thees, O., 2005. Applicability of the k-nearest neighbours (kNN-) method to predict the productivity of harvesting-basic considerations and first experiences. ALLGEMEINE FORST UND JAGDZEITUNG, 176(11-12), 189-200.

Lijewski, P., Merkisz, J., Fuć, P., Ziółkowski, A., Rymaniak, Ł., Kusiak, W., 2017. Fuel consumption and exhaust emissions in the process of mechanized timber extraction and transport. Eur J Forest Res 136 (1), 135–153. DOI: 10.1007/s10342-016-1015-2.

Nemestothy, N., Beguš, J., Klun, J., Krajnc N., Magaud, P., Rebolj, L., Triplat, M., 2014. Cost & benefits evaluation: HeProMo – the productivity estimation tool. In: Forest Logistic Planning Strategies. Good practices for the Alpine forests.

Neumann, M., & Schadauer, K., 2007. Holz-und Biomassenaufkommensstudie für Österreich– Hintergründe, Ausgangssituation und methodische Ansätze [wood and biomass resources study for Austria - background, situation and methodical approach]. Tagung der Sektion für Ertragskunde, 188-192.

Nordfjell, T., Athanassiadis, D., Talbot, B., 2003. Fuel consumption in forwarders. IJFE 14 (2), 11–20.

Marchi, E., Chung, W., Visser, R., Abbas, D., Nordfjell, T., Mederski, P.S., 2018. Sustainable Forest Operations (SFO): a new paradigm in a changing world and climate. Sci Total Environ 634, 1385–97. https://doi.org/10.1016/j.scitotenv.2018.04.084.

Millennium Ecosystem Assessment (MEA), 2005. Ecosystems and Human Well-being Synthesis. Island Press, Washington, DC. Available at https://www.millenniumassessment.org/documents/document.356.aspx.pdf (latest access 2019/07/30).

Mederski, P., 2006. Comparison of harvesting productivity and costs in thinning operations with and without midfield. Forest Ecol Manage 224 (3), 286–296. https://doi.org/10.1016/j.foreco.2005.12.042.

Murphy, G., Lyons, J., O'Shea, M., Mullooly, G., Keane, E., & Devlin, G., 2010. Management tools for optimal allocation of wood fibre to conventional log and bio-energy markets in Ireland: a case study. European journal of forest research, 129(6), 1057-1067.

Olschewski, R.; Sandström, C.; Kasymov, U.; Johansson, J.; Fürst, C.; Ring, I., 2018. Policy forum: challenges and opportunities in developing new forest governance systems: insights from the IPBES assessment for Europe and Central Asia. Forest Policy and Economics, 97, 175-179. doi: 10.1016/j.forpol.2018.10.007

Pandur, Z., Šušnjar, M., Zorić, M., Nevečerel, H., Horvat, D., 2015. Energy Return on Investment (EROI) of Different Wood Products. In: Zlatić, M. (Ed.), Precious Forests: Precious Earth. InTech, London, UK.

Schanz, H., 2004. Sustainable Forest management. In: Burley J, Evans J, Youngquist J, editors. Encyclopedia of Forest Sciences. Stadt. Amsterdam: Elsevier Academic Press.

Schweier, J., Ludowicy, C., 2020. Comparison of a cable-based and a ground-based system in flat and soil-sensitive area: a case study from south Baden in Germany. Forests 11(6), 611; https://doi.org/10.3390/f11060611.

Sirén, M., Aaltio, H., 2003. Productivity and Costs of Thinning Harvesters and Harvester-Forwarders. IJFE 14 (1), 39–48. DOI: 10.1080/14942119.2003.10702468.

Stadelmann, G., Temperli, C., Rohner, B., Didion, M., Herold, A., Rösler, E., & Thürig, E., 2019a. Presenting MASSIMO: a management scenario simulation model to project growth, harvests and carbon dynamics of Swiss forests. Forests, 10(2), 94.

Stadelmann, G., Temperli, C., Rohner, B., Didion, M., Herold, A., Rösler, E., & Thürig, E., 2019b. Forest Development Model MASSIMO. In Swiss National Forest Inventory–Methods and Models of the Fourth Assessment (pp. 265-279). Springer, Cham.

Thees, O., & Frutig, F., 2018. Holzernte-Produktivitätsmodelle “HeProMo” [Wood harvesting productivity models "HeProMo"]. Presentation, Freising, Germany. https://www.fvt.wzw.tum.de/fileadmin/pdf/U-Tag_2018_Vortraege/Unternehmertag_2018_01_Thees_Holzernte-Produktivitaetsmodelle.pdf

ThüringenForst, 2014. Kompendium zur Waldarbeit, Teil 15: Kalkulationsgrundlagen Rückung und mechanisierte Holzernte [Forest work compendium, part 15: calculation fundamentals skidding and mechanical wood harvesting]. Internal Document, first published 1994.

Triplat, M., Krajnc, N., 2020. Assessment of Costs in Harvesting Systems Using WoodChainManager Web-based Tool. Croat J For Eng 41(1), 49–57. DOI: https://doi.org/10.5552/crojfe.2020.583.

Uusitalo J., 2010. Introduction to Forest Operations and Technology. Hämeenlinna: JVP Forest Systems Oy; 2010.

Werner, F., 2017. Background report for the life cycle inventories of wood and wood based products for updates of ecoinvent 2.2. Retrieved from http://www.dflca.ch/inventories/Hintergrund/Werner_2017-report_wood_KBOB_2016.pdf
